# A Functional Magnetic Resonance Imaging Study of Pathophysiological Changes Responsible for Mirror Movements in Parkinson’s Disease

**DOI:** 10.1371/journal.pone.0066910

**Published:** 2013-06-25

**Authors:** Alice Poisson, Bénédicte Ballanger, Elise Metereau, Jérome Redouté, Danielle Ibarolla, Jean-Christophe Comte, Hélène Gervais Bernard, Marie Vidailhet, Emmanuel Broussolle, Stéphane Thobois

**Affiliations:** 1 Université de Lyon, Faculté de Médecine Lyon Sud Charles Mérieux, Lyon, France; 2 CNRS, UMR5229, Centre de Neuroscience Cognitive, Bron, France; 3 Hospices Civils de Lyon, Hôpital Neurologique Pierre Wertheimer, Service de Neurologie C, Lyon, France; 4 CERMEP Imagerie du vivant, Lyon, France; 5 Fédération de Neurologie, CRICM UMR-S UPMC/INSERM 975; CNRS UMR 7225, Hôpital de la Pitié-Salpêtrière, Paris, France; University of Minnesota, United States of America

## Abstract

Mirror movements correspond to involuntary movements observed in the limb contralateral to the one performing voluntary movement. They can be observed in Parkinson’s disease (PD) but their pathophysiology remains unclear. The present study aims at identifying their neural correlates in PD using functional magnetic resonance imaging. Ten control subjects and 14-off drug patients with asymmetrical right-sided PD were included (8 with left-sided mirror movements during right-hand movements, and 6 without mirror movements). Between-group comparisons of BOLD signal were performed during right-hand movements and at rest (p<0.005 uncorrected). The comparison between PD patients with and without mirror movements showed that mirror movements were associated with an overactivation of the insula, precuneus/posterior cingulate cortex bilaterally and of the left inferior frontal cortex and with a deactivation of the right dorsolateral prefrontal cortex, medial prefrontal cortex, and pre-supplementary motor area and occipital cortex. These data suggest that mirror movements in Parkinson’s disease are promoted by: 1- a deactivation of the non-mirroring inhibitory network (dorsolateral prefrontal cortex, pre-supplementary motor area); 2- an overactivation of prokinetic areas (notably the insula). The concomitant overactivation of a proactive inhibitory network (including the posterior cingulate cortex and precuneus) could reflect a compensatory inhibition of mirror movements.

## Introduction

Mirror movements (MM) correspond to involuntary movements observed during voluntary activity in contralateral homologous body regions (see for review: [1]). Physiological MM are frequently noted during childhood and usually vanish in adulthood because of physiological brain maturation [2]. When persisting in adulthood, MM are usually pathological and may reflect an underlying disease such as Parkinson’s disease (PD). In PD, MM are unilateral and affect the less affected hemibody during voluntary movements of the most affected limb, whether patients are treated or not [3], [4]. The prevalence of MM in PD is greater in the early and mid stages but these abnormal movements can be seen up to 5 years after disease onset [3]–[6]. MM usually predominate at the upper limbs [4]. The occurrence of MM is correlated with the severity of lateralized motor symptoms but also with levodopa-responsiveness [3]–[6]. MM are not disabling but of interest as they could be considered as the “tip of the iceberg” of complex brain circuitry reorganization in PD. Thus, studying their mechanisms could shed light on the pathophysiology of PD per se.

Pathophysiology of MM depends on the underlying pathology. An overrepresentation of the direct pyramidal pathway is suspected in some genetic diseases [7], [8]. An abnormal activation of the primary motor cortex (M1) ipsilaterally to the voluntary movement could also explain the occurrence of MM during post-stroke recovery but in PD as well [9]–[13]. Furthermore, other studies have suggested that MM could be induced by a dysfunction of a non-mirroring network including the SMA [14]–[16]. All these mechanisms are not mutually exclusive and may coexist to explain MM in PD.

The purpose of the present study was to better understand the pathophysiology of MM in PD using functional magnetic resonance imaging (fMRI) technique in PD patients with and without MM.

## Materials and Methods

### Subjects

Three groups of subjects participated in the study. Eight right-handed PD patients with clinically evident left-hand MM during voluntary movements performed with the right, most akinetic hand were included (PD+MM). MM were defined as involuntary left hand movements mimicking voluntary movements of the contralateral hand through the activation of homologous muscles. Six right-handed PD patients without MM were enrolled (PD-MM). Ten right-handed healthy subjects were also recruited by public announcement. These healthy subjects were carefully examined by one of the clinician (ST, AP) in charge of the study to exclude any abnormalities of the neurological examination and to ensure that they did not exhibit any MM during right hand movements. All PD patients presented asymmetrical Parkinsonian signs predominating on the right hemibody. Asymmetry of motor symptoms was determined by the hand tapping test and lateralized UPDRS motor score. The hand tapping test consisted in tapping alternatively and as fast as possible with the right or the left index on two buttons located in front of the patient and separated by 30 cm. The total number of taps made in 30 sec was recorded. Motor asymmetry was defined, in the present study, by, at least, a 10% difference between the two sides on the hand-tapping test or a 2-point difference on item 25 (pronosupination movements) of the motor section of the Unified Parkinson Disease Rating Scale (UPDRS) [17], [18]. MM severity was assessed with the Woods and Teuber scale during different movements of the right hand and wrist (prono-supination, hand opening and closing, index to thumb opposition and wrist flexion extension movements) [19]. MM severity was quoted using the following scale: 0, no clear imitative movement; 1, barely discernible repetitive movement; 2, slight mirror movements; 3, sustained mirror movements; 4, movement equal to that expected for the intended hand [19]. Total score for each patient is comprised between 0 and 16. After patients had been selected on a clinical basis, the presence or absence of MM was confirmed by a 10 kHz sampled rate surface electromyogram (EMG) of both flexor pollicis longus.

Exclusion criteria for the 3 groups of subjects were: other neurological diseases, Mini-Mental State <24, ferromagnetic implanted material, claustrophobia and pregnancy. For PD patients, severe tremor in off-medication condition constituted another exclusion criteria. Clinical characteristics of PD patients and controls are summarized in Table 1.

**Table 1:** Clinical characteristics of PD patients and controls.

		Age (years)(mean +/− SD)	Disease duration (years) (mean +/SD)	UPDRS III score Off medication (mean +/− SD)	Right-sided Motor UPDRS III score (mean +/− SD)	MM score	Levodopa Eq (mg/day)
PD+MM	n = 8 5M, 3F	59 (7.7)	4.5 (1.7)	17.7 (4.6)	9.75 (3)	3.9 (3.3)	339 (165)
PD-MM	n = 6 4M,2F	65 (10)	5.4 (4,6)	16 (5.1)	6.6 (0.5)	0	458 (361)
Controls	n = 10 4M,6F	53.6 (8.5)	NA	NA	NA	0	NA
p-value		0.24	0.66	0.58	0.02*		0.48

Levodopa Eq = levodopa equivalent: 100 mg L-dopa = 10 mg Bromocriptine = 5mg Ropinirole = 1 mg Pergolide = 50 mg Piribedil = 1 mg Pramipexole [20]; SD: Standard Deviation;

* p<0.05.

On the day of the fMRI, PD patients were off antiparkinsonian drugs for at least 12 hours.

This study was approved by the local research ethics committee : Comité de Protection des Personnes SUD-EST IV, Lyon, France. All subjects participated after the aim of the study and the nature of the procedure had been fully explained and they had signed an informed consent according to the Declaration of Helsinki.

### Tasks

fMRI images acquisition was performed during the execution of a motor task (M) and at rest (R). The motor task (M) consisted in repetitive index to thumb opposition movements. The motor task was externally cued by a 1 Hz auditory stimulus and performed with the right (akinetic) hand (that induced MM in the PD+MM group of patients). At-rest (R) subjects were lying on the bed of the MRI, looking at a blue screen, without moving. During MRI acquisition, a surface EMG (BIOPAC MP150) was performed to check for presence (or absence) of MM during the execution of the task.

A 10 Hz to 500 Hz Band-Pass Finit Impulse Response Filter (FIR) was used and the instantaneous power of the signal was determined. Finally, EMG power during movements versus rest was compared in order to remove the fMRI artefacts.

### Functional Magnetic Resonance Imaging Scanning

Subjects were scanned using a 1.5-Tesla Siemens Magnetom Sonata scanner at the CERMEP- Imaging Centre (Lyon, France). Echoplanar imaging (EPI) settings were as follows: repetition time: 3.0 s, matrix size: 64×64 voxels, voxel size: 2.0×2.0×2.0 mm, echo time: 60 ms, 26 axial CACP oriented slices acquired in ascending interleaved order. Functional images were acquired in two runs, each lasting 6 minutes. Each run consisted in the alternation of task and rest conditions.

### Data Analysis

Data were imported from DICOM format, transformed in the “nifti” file format and then processed in MATLAB 7 (MathWorks, Nack, MA, USA) using the Statistical Parametric Mapping software (SPM 5; Wellcome Department of Cognitive Neurology, MRC Cyclotron Unit, London, UK). The first four frames of each run were suppressed to account for magnetic saturation effects. Images were realigned to the fifth frame for motion correction, then normalized to the SPM5 template and smoothed with a Gaussian kernel of 8×8×8 mm. For each subject, a separate linear general model was constructed including two regressors of interest defined by the movement of the right hand and the rest condition. This model was convolved with the canonical hemodynamic response function (HRF) and the parameters estimated were drawn into statistical parametric t-maps reflecting the correlation between BOLD signal and the hand movement. Individual results were then entered into a 2nd-level random effect group analysis through applying a 1-sample *t-*test to the first-level t-maps resulting from individual correlation analyses.

### Statistical Analysis

#### Within-group Analysis

Increase of BOLD signal during right-hand movements compared to rest was assessed using the following contrast (M – R) in each group. At the group level, for the patients with MM, a multiple regression analysis was performed to search for brain regions in which the severity of MM (based on the Wood and Teuber score) explains the variability in the cerebral activity.

#### Between-group Comparisons

Brain activation abnormalities related to PD independently of the presence of MM were determined by comparing healthy subjects and PD patients without MM. Increase and decrease of brain activation during right hand movements in PD-MM patients relative to healthy subjects were assessed respectively using the following contrasts: (M – R)_PD-MM_ - (M – R)_controls_ and (M – R)_controls_ - (M – R)_PD-MM_.

Brain activation abnormalities specifically associated with MM were assessed by comparing PD+MM and PD-MM patients. Increase of brain activation related to MM was determined using the following contrast: (M – R)_PD+MM_ - (M – R)_PD-MM_. The opposite contrast ((M – R)_PD-MM_ - (M – R)_PD+MM_ ) revealed the decrease of brain activation associated with MM.

Global differences in BOLD signal were covaried out for all brain voxels. Comparisons across conditions were made using *t* statistics with appropriate linear contrasts (see above). Only voxels exceeding a threshold of an uncorrected p-value ≤0.005 with a minimal cluster size of 10 voxels were considered as significant. All coordinates reported are derived from the MNI Space Utility toolbox (MSU http://www.ihb.spb.ru/~pet_lab/MSU/MSUMain.html).

## Results

### Clinical Findings

The severity of MM was mild to moderate in all the PP+MM patients. The Wood and Teuber scale score was indeed comprised between 2 and 7 on a 16 points scale. No difference between the two groups of patients was noted for disease duration, antiparkinsonian treatment and global severity of motor signs. However, the lateralized UPDRS III subscore showed that PD-MM patients were less severely affected on the right side than PD+MM patients.

### Functional Imaging Findings

#### 1) Control group

During movement of the right hand, increase of BOLD signal was observed in the left M1 (Brodmann area (BA) 4), SMA (BA 6), superior temporal gyrus (BA 22) and primary somatosensory cortex (BA 3) as well as in the right inferior parietal lobe (BA 40).

#### 2) PD patients without MM (PD-MM)

During movement of the right hand, increase of BOLD signal was noted in the left M1 (BA 4), SMA (BA 6), inferior parietal lobe (BA 40), the right pre-SMA, lateral premotor cortex (BA 6), inferior temporal gyrus, (BA 20), bilateral superior temporal gyrus (BA 22) as well as in the vermis.

#### 3) PD patients with MM (PD+MM)

During movement of the right hand, increase of BOLD signal was observed in right primary somatosensory cortex (BA 3) and inferior frontal gyrus (BA 47), the left inferior parietal lobe (BA 40), caudate nucleus, as well as bilaterally in the insula (BA 13), ventral anterior cingulate cortex (BA 24/32), posterior cingulate cortex (PCC) (BA 31) and inferior temporal lobe (BA 20).

#### 4) Changes in BOLD activity related to PD

Overactivations during right-hand movements in PD-MM group compared to controls were observed in the right parahipoccampal gyrus (BA 36), lateral premotor cortex (BA 6), DLPFC (BA 46), occipital cortex (BA 18), and in the left M1 (BA 4), inferior parietal lobe (BA 40), head of caudate nucleus, as well as bilaterally in cerebellar hemispheres. In contrast, right-hand movements produced BOLD signal decrease in the right primary sensory cortex (BA 3) and PCC (BA 31), as well as in the precuneus bilaterally (BA 7) in PD-MM group relative to controls. These results are detailed in **Table 2**.

**Table 2:** Changes of BOLD signal related to Parkinson’s disease.

Area	Left/Right		BA	Stereotactic Coordinates (Regional Maximal)					T value	P uncorr	Cluster Size
				x		y		z			
***Increased BOLD signal related to PD (PD-MM >controls)***											
Occipital cortex	R	18		2	−100		8		4.18	>0.0001	40
Inferior parietal lobe	L	40		−66	−34		30		4.01	0.001	28
Parahippocampal gyrus	R	36		28	−12		−28		3.75	0.001	16
Lateral premotor cortex	R	6		36	−2		62		3.65	0.001	22
DLPFC	R	46		38	60		20		3.62	0.001	13
M1/Primary motor cortex	L	4		−26	−34		76		3.60	0.001	16
Caudate nucleus	L			−24	0		−12		3.48	0.002	20
Cerebellum	L			−4	−78		−36		4.51	0.000	37
Cerebellum	R			14	−84		−28		4.48	0.000	62
***Decreased BOLD signal related to PD (control >PD-MM)***											
Post central gyrus	R	3		60	−32		52		4.68	>0.0001	30
PCC	R	31		8	−62		26		4.44	>0.0001	47
Precuneus	R	7		12	−56		60		3.78	0.001	12
Precuneus	L	7		−18	−64		26		3.55	0.001	12

Abbreviations : L = left; R = right; BA = Brodmann’s area

#### 5) Changes in BOLD activity related to MM

**Figure 1:**
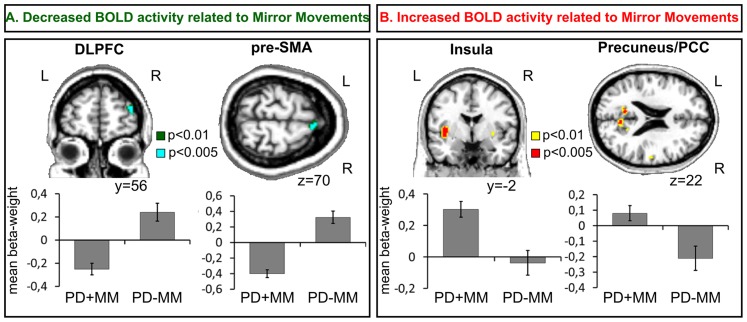
BOLD signal changes related to Mirror Movements. **A:** Reduction of activation during MM (PD+MM <PD-MM). **B:** Increase of activation during MM (PD+MM >PD-MM). Graphs show the regionally averaged beta weights across patients from each group. Error bars indicate inter-patient standard error of the mean (SEM). L = left; R = right.

The presence of MM was associated with BOLD signal decrease in the right DLPFC (BA 10), medial prefrontal cortex (mPFC, BA 9)**,** pre-SMA (BA 6), and in medial occipital gyrus (BA 18/19). In addition, increases of BOLD signal were observed in the left inferior frontal gyrus (BA 47) and PCC (BA 31), in the right precuneus (BA 7), and bilaterally in the insula (BA 13). We did not observe significant difference of activation of ispilateral to the movement primary motor cortex. Furthermore, no correlation between the severity of MM and abnormalities of brain activation profile was disclosed. These results are presented in **Table 3** **and** **Figure 1**.

**Table 3:** Changes of BOLD signal related to Mirror Movements.

Area	Left/Right	BA	Stereotactic Coordinates (Regional Maximal)			T value	P uncorrected	Cluster Size
			x	y	z			
***Increased BOLD signal related to MM (PD+MM>PD-MM)***								
Insula	L	13	−40	−4	2	4.09	0.001	165
Insula	R	13	32	−4	−2	3.47	0.002	10
Precuneus	R	7	4	−62	24	3.62	0.002	25
Inferior Frontal Gyrus	L	47	−42	20	−12	3.46	0.002	41
PCC	L	31	−8	−56	22	3.38	0.002	10
***Decreased BOLD signal related to MM (PD-MM>PD+MM)***								
DLPFC	R	10	42	56	16	4.08	0.001	33
mPFC	R	9	12	62	30	3.75	0.001	16
Pre-SMA	R	6	8	16	70	3.72	0.001	19
Medial occipital gyrus	R	19	44	−82	−4	3.67	0.001	38

Abbreviations : L : left, R : right, BA : Brodmann’s Area

## Discussion

The present study demonstrates that MM in PD are mostly linked to a deactivation of pre-SMA, mPFC, and DLPFC regions concomitantly with an overactivation of the insula, precuneus, PCC and inferior frontal gyrus.

Before discussing the results related to MM pathophysiology, it should be briefly mentioned that widespread overactivations were observed during motor execution in patients with PD without MM. Indeed, PD patients recruit a large cerebellar-parietal-premotor-motor network involving regions dedicated both to motor execution and preparation [21]. This fits well with the recruitment of the so-called cerebellar-parietal-premotor accessory motor pathway, which is supposed to be compensatory in PD when fronto-mesio-striatal loops fail [21]–[23]. The overactivation of DLPFC and left head of caudate nucleus observed in our PD patients could as well correspond to a compensatory recruitment of the associative circuit [24].

### Functional Basis of Mirror Movements in Parkinson’s Disease

The present study suggests that MM occurrence in PD is due to a combination of, on the one hand, a deficit in inhibitory mechanisms and, on the other hand, an excessive recruitment of brain areas.

Indeed, a decrease of BOLD signal was found in PD patients with MM compared to patients without in the pre-SMA and DLPFC, two regions involved in inhibitory processes [25]. Interestingly, Chan and colleagues reported the occurrence of MM after ischemic lesion of a large area including the SMA, but also the mPFC and anterior cingulate cortex [14]. Furthermore, the SMA belongs to a non-mirroring system aiming at physiologically suppressing MM [14], [16]. Thus, its dysfunction could play a major role in MM occurrence in PD. The DLPFC is also part of a neural network supporting both selection and suppression of motor responses [26]. Hence, the DLPFC could also be involved in the physiological inhibition of MM. Overall, MM in PD could be due to a deficit of activation of areas involved in reactive inhibitory processes.

On the other hand, in comparison to patients without MM, PD patients with MM clearly displayed a greater and less lateralized recruitment of numerous brain regions during motor execution. It is tempting to speculate that this abnormal increase of brain activation could also play a role in the occurrence of MM in PD. Among those areas, the role of the insula appears important. Interestingly, some functional imaging studies have shown, during post-stroke motor recovery, an increased activation of this region when movement was performed with the paretic hand. Unfortunately, the authors did not investigate the presence of MM [27]. Nevertheless, this suggests a compensatory (i.e prokinetic) role of the insular region possibly through the archaic insular sensori-motor cortex [27]. The present data could suggest that the insula overactivation, aiming in the first instance to improve motor performance, could as well, because of its bilateral recruitment, lead to MM. One of the possible mechanisms of this brain overflow could be a reduced transcallosal inhibition or increased transcallosal facilitation leading to a less lateralized brain activation, which, in turn could favor the occurrence of MM [13].

Apart from the insula, the presence of MM was also associated with an increased BOLD signal in the precuneus/PCC node, which is part of the so-called “default brain network”, and is involved in proactive inhibitory processes [28]. Boulinguez and colleagues have recently proposed a “proactive inhibition” model in which the precuneus/PCC play a central role to avoid inappropriate behavior [29]–[31]. Thus, regarding MM pathophysiology in PD, the precuneus/PCC node overactivation could be interpreted as an attempt to inhibit these abnormal and involuntary movements. The overactivation of the left inferior frontal cortex could be viewed in the same way [32]. Indeed, the ventrolateral prefrontal cortex plays a crucial role in response inhibition and interference suppression [33].

The lack of correlation between MM severity and abnormalities of brain activation pattern was probably due to the small sample size and to a relative homogeneous severity of MM.

Altogether, these results support a complex reorganization of brain activation pattern responsible for the occurrence of MM in PD patients. Deficit of areas primarily involved in MM inhibition and excessive recruitment of prokinetic areas could represent the primum movens of MM. In turn, the recruitment of the brain default network could be interpreted as a compensatory inhibition of MM.
